# 4th Annual Mediclinic Middle East Research Conference: Erratum

**DOI:** 10.1097/MD.0000000000028079

**Published:** 2021-12-03

In “4th Annual Mediclinic Middle East Research Conference”,^[1]^ which published in Volume 100, Issue 33 of *Medicine*, Dr Kassem El-Shunnar, Consultant Neurosurgeon- Mediclinic City Hospital, was not included in the abstract, “NECK PAIN RELATED TO SMART PHONE AND LAPTOP USE.” This has been corrected.
